# Biological Properties of Fucoxanthin in Oil Recovered from Two Brown Seaweeds Using Supercritical CO_2_ Extraction

**DOI:** 10.3390/md13063422

**Published:** 2015-05-29

**Authors:** Saravana Periaswamy Sivagnanam, Shipeng Yin, Jae Hyung Choi, Yong Beom Park, Hee Chul Woo, Byung Soo Chun

**Affiliations:** 1 Department of Food Science and Technology, Pukyong National University, 45 Yongso-ro, Nam-gu, Busan 608-737, Korea; E-Mails: saravana_ps@pknu.ac.kr (P.S.S.); dolen1900@163.com (S.Y.); 2 Department of Chemical Engineering, Pukyong National University, 365 Sinseon-ro, 599-1 Daeyeon-3dong, Nam-gu, Busan 608-737, Korea; E-Mails: jhchoe@pknu.ac.kr (J.H.C.); applechem@pknu.ac.kr (Y.B.P.); woohc@pknu.ac.kr (H.C.W.)

**Keywords:** brown seaweed, *Sargassum horneri*, *Saccharina japonica*, supercritical CO_2_ fucoxanthin, fatty acid, antioxidant, antimicrobial, antihypertensive

## Abstract

The bioactive materials in brown seaweeds hold great interest for developing new drugs and healthy foods. The oil content in brown seaweeds (*Saccharina japonica* and *Sargassum horneri*) was extracted by using environmentally friendly supercritical CO_2_ (SC-CO_2_) with ethanol as a co-solvent in a semi-batch flow extraction process and compared the results with a conventional extraction process using hexane, ethanol, and acetone mixed with methanol (1:1, v/v). The SC-CO_2_ method was used at a temperature of 45 °C and pressure of 250 bar. The flow rate of CO_2_ (27 g/min) was constant for the entire extraction period of 2 h. The obtained oil from the brown seaweeds was analyzed to determine their valuable compounds such as fatty acids, phenolic compounds, fucoxanthin and biological properties including antioxidant, antimicrobial, and antihypertension effects. The amounts of fucoxanthin extracted from the SC-CO_2_ oils of *S. japonica* and *S. horneri* were 0.41 ± 0.05 and 0.77 ± 0.07 mg/g, respectively. High antihypertensive activity was detected when using mixed acetone and methanol, whereas the phenolic content and antioxidant property were higher in the oil extracted by SC-CO_2_. The acetone–methanol mix extracts exhibited better antimicrobial activities than those obtained by other means. Thus, the SC-CO_2_ extraction process appears to be a good method for obtaining valuable compounds from both brown seaweeds, and showed stronger biological activity than that obtained by the conventional extraction process.

## 1. Introduction

Algae can be divided into two groups including macro-algae, or seaweeds and micro-algae. Similar to true plants, seaweeds are photosynthetic and form basic biomasses in intertidal zones. Approximately, 9000 seaweed species have been identified. On the basis of their pigmentation, these species have been broadly classified into three main groups of brown (Phaeophyta), red (Rhodophyta), and green (Chlorophyta) seaweeds [1]. Seaweeds contain several physiologically bioactive compounds with important economical relevance including polysaccharides, iodine organic products, macro- and micro-elements, vitamins, and unsaturated fatty acids (FAs) [2]. Brown seaweeds, the second-most abundant group of marine algae, include approximately 2000 species. Among them, *Sargassum* spp., *Laminaria* spp., *Ascophyllum* spp., *Fucus* spp., and *Turbinaria* spp. are most commonly used at the industrial level [3]. Because seaweeds lack many of the distinct organs found in terrestrial plants, the entire plant is available as a biomass source. A significant amount of attention has been paid recently to seaweeds as effective biomass sources because of their high CO_2_ absorption rate relative to that of terrestrial plants [1]. Seaweeds are of emerging interest in biomedical areas mainly due to their content of bioactive substances that show high potential as antioxidants, antimicrobials, anticoagulants, antithrombotics, and anti-inflammatories for the treatment of several diseases, in addition to their anti-tumor and anti-viral properties [4,5,6]. Therefore, seaweeds have been explored as sources of food, medicine, cosmetics, fertilizer, feed, and bio-energy [7].

The antioxidant sources of seaweeds are related mainly to their polyphenol content, particularly phlorotannins, which are the largest group of polyphenols in brown seaweed [7]. Seaweed lipids have drawn increased interest due to their health benefits. Brown seaweed lipids contain many types of bioactive compounds such as omega-3 polyunsaturated (PUFAs), omega-6, arachidonic acid, fucoxanthin, fucosterol, and some polyphenols. Among these compounds, fucoxanthin, a major carotenoid in brown seaweed, is regarded as a nutraceutical compound specific to brown seaweed lipids because it shows several physiological effects based on unique molecular mechanisms [8]. Therefore, brown seaweed represents a highly functional lipid source. However, because of the high level of omega-3 PUFAs such as eicosapentaenoic acid (20:5*n*-3) and stearidonic acid (18:4*n*-3), brown seaweed lipids may be susceptible to oxidation [1].

Fucoxanthin, a member of the carotenoid family, is abundant in edible brown algae and contributes to more than 10% of the estimated total production of carotenoids in nature. This compound plays a role in light harvesting and energy transfer and thus helps marine brown alga survive in shallow coastal waters by offering efficient photosynthesis for acclimatization to their environment [9]. Although less attention has been paid to the physiological effects of carotenoids in seaweeds, fucoxanthin has recently attracted high attention due to its strong antioxidant properties against the effects of cancer, hypertension, obesity, and inflammation [10].

Hypertension is considered to be the most common chronic disease and a major risk factor for cardiovascular disease, a main cause of death worldwide [11]. Angiotensin-I converting enzyme (ACE) regulates blood pressure function by converting angiotensin-I into a potent vasoconstrictor, angiotensin-II, and catalyzing the degradation of a potent vasodilator, bradykinin [12]. ACE inhibitors (ACE-I), a new class of antiangiotensive drugs, are effective in inhibiting the formation of angiotensin-II [13]. Synthetic ACE-I has been developed for antihypertension drugs including captopril, enalapril, alacepril, and lisinopril, which are widely used in the treatment of hypertension and heart failure in humans. However, some side effects caused by these synthetic drugs have been reported [12]. Many ACE-inhibitory peptides have been discovered in the enzymatic hydrolysates of various protein-rich foods such as soy sauce, fish sauce, sake, soybeans, and milk. Among various sources, marine organisms have been widely used in the search for ACE-I peptides [13]. Hence, scientists have focused special attention on marine algae to identify and evaluate natural antihypertension compounds. The amino acid heterogeneity of proteins is very high among seaweeds even in those containing low amounts of protein. Therefore, it is believed that the evaluation of algal species is a promising way to find new anti-hypertensive compounds [11].

The aim of this study is to extract the oil from two brown seaweeds, *S. japonica* and *S. horneri*, by using environmental friendly SC-CO_2_ extraction and in addition to that by conventional solvents. The obtained oil was investigated to determine its composition of FAs, fucoxanthin, and total phenolics in addition to antioxidant, antimicrobial, and antihypertension activities.

## 2. Results and Discussion

### 2.1. Conventional Solvent Extraction

The efficiencies of oil extracted by using three different solvents including hexane, ethanol, and acetone mixed with methanol (1:1, v/v) was checked by examining their different polarities. As shown in Table 1, various oil yields were produced by the various solvents. The extracted oil contents in *S. japonica* and *S. horneri* were 1.19 ± 0.21 and 1.29 ± 0.05 g/100 g dry weight (DW) when acetone mixed with methanol was used as the solvent, 1.24 ± 0.06 and 1.42 ± 0.08 g/100 g DW when hexane was used, and 1.22 ± 0.12 and 1.36 ± 0.14 g/100 g DW when ethanol was used, respectively. These results indicate that acetone mixed with methanol was the most efficient extraction solvent. Furthermore, the recovery of acetone–methanol was the highest of the solvents in both the seaweeds, and *S. horneri* had the higher oil yield.

### 2.2. Extraction of Oil Using SC-CO_2_ with Ethanol as Co-Solvent

In a previous study, the extraction of lipids and fucoxanthin was reported to be very low from brown seaweed when pure SC-CO_2_ was used; however, the yield was increased when using a co-solvent [14,15]. In the present study, we evaluated the effects of SC-CO_2_ with ethanol as a co-solvent in the extraction of oil from brown seaweeds at operational condition of 250 bar and 45 °C. The oil contents obtained from *S. japonica* and *S. horneri* through this process were 1.09 ± 0.56 and 1.41 ± 0.15 g/100 g DW, respectively (Table 1). Therefore, extraction experiments using SC-CO_2_ with ethanol as a co-solvent can yield similar oil content through an environmental friendly extraction process.

Conde *et al.* [15] reported that under the influence of SC-CO_2_ modified with 10% ethanol, the extract yield from *S. muticum* was significantly improved; the yields of total oil and fucoxanthin were up to three and 90 times higher, respectively.

**Table 1:** Extraction yield and fucoxanthin content of brown seaweeds from various extracts.

Solvent	Extraction Yield (g/100 g DW)		Fucoxanthin Content (mg/g)	
	*S. japonica*	*S. horneri*	*S. japonica*	*S. horneri*
SC-CO_2_ + Ethanol	1.09 ± 0.56 ^a^	1.41 ± 0.15 ^b^	0.41 ± 0.05 ^a^	0.77 ± 0.07 ^a^
Acetone mix methanol	1.19 ± 0.21 ^a^	1.29 ± 0.05 ^a^	0.48 ± 0.10 ^b^	0.71 ± 0.05 ^b^
Hexane	1.24 ± 0.06 ^a^	1.42 ± 0.08 ^c^	0.16 ± 0.01 ^a^	0.05 ± 0.03 ^c^
Ethanol	1.22 ± 0.12 ^a^	1.36 ± 0.14 ^c^	0.12 ± 0.02 ^a,b^	0.08 ± 0.05 ^c^

Values are expressed as mean ± SD. Different letters indicate significant differences (*p* < 0.05) according to Tukey’s multiple range test.

### 2.3. Measurement of FA Composition and Fucoxanthin Content

The FA compositions of the obtained oil from different extraction systems including hexane, ethanol, acetone–methanol extract, and SC-CO_2_ with ethanol from brown seaweeds determined by gas chromatography (GC) are shown in Table 2. The percentages of the total saturated FAs (SFAs) were high in *S. horneri* extracts, whereas those of monounsaturated FAs (MUFAs) were higher in the *S. japonica* extracts. In *S. horneri* extracts, palmitic acid (C16:0) was found in high amounts ranging from 180.70 ± 1.80 to 233.20 ± 2.90 mg/g in extracted oil, whereas more elaidic acid was found in *S. japonica* extracts, from 168.90 ± 0.20 to 340.10 ± 0.10 mg/g in extracted oil. Important PUFAs such as eicosapentaenoic acid (EPA; C20:5*n*3) were present in high amounts. In *S. horneri*, 80.80 ± 0.90 mg/g in extracted oil of EPA was found in the SC-CO_2_ extract, and 70.60 ± 0.10 mg/g in extracted oil was in the acetone–methanol extract of *S. japonica*. No docosahexaenoic acid (DHA; C22:6*n*3) was found in the oil of either brown seaweed with the various solvents used in this experiment. Among the solvent systems, the total FA compositions were high in the SC-CO_2_ extract and in acetone–methanol extract. Terasaki *et al.* [16] reported that 9.70% EPA was found in *S. horneri*,and Dawczynski *et al.* [17] reported levels of 16.2% ± 8.90% in *Laminaria* sp. The lower amounts obtained from our experiments could be attributed to seasonal variation and continental location, which can dramatically alter the composition.

Seaweed products represent an important source of long-chain polyunsaturated FAs (LC-PUFA) that are fundamental for the formation of important structural lipids and elements of cell membranes. In addition, these LC-PUFA are precursors of eicosanoids, which influence inflammation processes and immune reactions [18]. Although these two classes of PUFA have opposing physiological functions, their balance is important for normal growth and development. These FAs are beneficial for the prevention of cardiovascular diseases and other chronic diseases such as diabetes, hypertension, and autoimmune diseases. The European Nutritional Societies have recommended that human diet include an *n*-6:*n*-3 ratio of 5:1 for promoting good health [19]. Thus, consumption of seaweed products can contribute to improvement in the dietary supply of *n*-3 FA. Intake of foods rich in *n*-3 LC-PUFA can have a positive influence on the composition of blood lipids and can therefore be used for the prevention of arteriosclerosis [20].

**Table 2:** Fatty acid composition of *S. japonica* and *S. horneri* from various extracts.

Fatty acid compositions		*S. japonica* (mg/g in Extracted Oil)				*S. horneri* (mg/g in Extracted Oil)			
		SC-CO_2_ + Ethanol	Acetone mix methanol	Hexane	Ethanol	SC-CO_2_ + Ethanol	Acetone mix methanol	Hexane	Ethanol
Saturated fatty acid (SFA)	C10:0	N.D	N.D	N.D	N.D	49.1 ± 0.70	51.4 ± 0.50	211.7 ± 3.1	N.D
	C11:0	N.D	N.D	N.D	N.D	N.D	8.5 ± 0.30	N.D	250.10 ± 3.50
	C12:0	N.D	N.D	N.D	N.D	17.4 ± 0.10	20.1 ± 0.20	27.9 ± 0.30	N.D
	C13:0	14.10 ± 1.0	N.D	N.D	N.D	34.3 ± 0.50	29.90 ± 0.20	23.00 ± 0.50	N.D
	C14:0	141.40 ± 8.00	123.8 ± 1.00	130.50 ± 1.40	114.80 ± 0.20	75.50 ± 0.80	65.80 ± 0.40	13.00 ± 0.30	38.60 ± 0.40
	C15:0	4.60 ± 0.10	10.20 ± 0.10	14.50 ± 0.20	4.30 ± 0.10	41.60 ± 0.50	52.40 ± 0.40	21.20 ± 0.40	16.30 ± 0.30
	C16:0	N.D	N.D	N.D	N.D	214.80 ± 3.40	185.40 ± 2.40	180.70 ± 1.80	233.2 ± 2.90
	C17:0	N.D	N.D	N.D	18.40 ± 0.10	N.D	20.10 ± 0.10	22.50 ± 0.60	N.D
	C18:0	N.D	N.D	N.D	N.D	17.10 ± 0.20	19.50 ± 0.20	26.00 ± 0.40	N.D
	C20:0	N.D	N.D	48.10 ± 0.10	51.70 ± 0.20	8.50 ± 0.10	12.10 ± 0.20	8.60 ± 0.10	29.80 ± 0.40
	C21:0	29.80 ± 0.10	107.10 ± 0.10	91.90 ± 1.00	102.70 ± 0.50	62.30 ± 0.80	61.10 ± 0.60	44.30 ± 0.40	39.30 ± 0.50
	C23:0	N.D	N.D	7.20 ± 0.10	5.80 ± 0.10	N.D	7.60 ± 0.20	N.D	11.30 ± 0.40
Total SFA		189.90 ± 9.20	241.10 ± 1.20	292.20 ± 2.80	297.70 ± 1.20	520.60 ± 7.10	533.90 ± 5.70	578.90 ± 7.90	618.60 ± 8.40
Monounsaturated fatty acid (MUFA)	C15:1	252.70 ± 0.10	179.80 ± 0.10	178.00 ± 1.00	200.90 ± 0.10	11.30 ± 0.20	15.90 ± 0.30	28.40 ± 0.50	N.D
	C14:1	5.00 ± 0.10	29.90 ± 0.00	8.60 ± 0.50	21.30 ± 0.40	16.60 ± 0.20	21.40 ± 0.30	21.10 ± 0.20	56.40 ± 0.50
	C16:1	18.70 ± 0.10	45.30 ± 0.10	45.80 ± 0.10	40.10 ± 0.70	26.90 ± 0.40	23.10 ± 0.20	13.10 ± 0.30	56.00 ± 0.80
	C17:1	26.90 ± 0.10	29.60 ± 0.40	10.20 ± 0.10	19.60 ± 0.10	6.20 ± 0.10	29.60 ± 0.40	37.90 ± 0.70	N.D
	C18:1*n*9c	N.D	N.D	N.D	N.D	122.90 ± 0.50	112.40 ± 0.70	89.80 ± 0.70	83.70 ± 1.10
	C18:1*n*9t	340.10 ± 0.10	182.90 ± 0.50	177.50 ± 1.00	168.90 ± 0.20	16.90 ± 0.20	18.50 ± 0.50	23.40 ± 0.30	N.D
	C22:1*n*9	N.D	N.D	N.D	N.D	N.D	8.30 ± 0.20	N.D	N.D
Total MUFA		644.30 ± 0.50	467.50 ± 1.10	420.10 ± 2.70	450.80 ± 1.50	200.80 ± 1.60	229.20 ± 2.60	213.70 ± 2.70	196.10 ± 2.40
Polyunsaturated fatty acid (PUFA)	C18:2*n*6c	10.40 ± 0.10	53.80 ± 0.20	78.70 ± 0.10	83.50 ± 0.20	7.30 ± 0.10	12.50 ± 0.10	11.70 ± 0.20	38.60 ± 0.40
	C18:2*n*6t	4.10 ± 0.10	N.D	N.D	N.D	64.30 ± 0.50	70.10 ± 0.40	20.60 ± 0.40	N.D
	C18:3*n*6	23.20 ± 0.10	47.80 ± 0.10	45.00 ± 0.10	45.90 ± 0.50	24.60 ± 0.30	16.90 ± 0.50	9.50 ± 0.10	N.D
	C20:2	23.50 ± 0.10	56.40 ± 1.00	N.D	N.D	10.50 ± 0.10	18.70 ± 0.30	10.60 ± 0.20	22.0 ± 0.40
	C18:3*n*3	N.D	N.D	N.D	N.D	N.D	4.50 ± 0.10	5.10 ± 0.20	30.20 ± 0.60
	C20:3*n*6	27.80 ± 0.20	62.80 ± 0.50	107.00 ± 1.40	104.80 ± 0.80	65.80 ± 0.90	64.50 ± 0.50	66.70 ± 0.70	60.10 ± 0.80
	C22:2	N.D	N.D	N.D	N.D	13.90 ± 0.30	9.50 ± 0.40	8.00 ± 0.10	N.D
	C20:5*n*3	57.70 ± 0.40	70.60 ± 0.10	57.00 ± 0.20	17.30 ± 0.20	80.80 ± 0.90	54.70 ± 1.20	75.20 ± 0.80	34.40 ± 0.50
	C22:6*n*3	N.D	N.D	N.D	N.D	N.D	N.D	N.D	N.D
Total PUFA		146.70 ± 1.00	291.40 ± 1.90	287.70 ± 1.80	251.50 ± 1.70	267.20 ± 3.10	251.40 ± 3.50	202.30 ± 2.70	185.30 ± 2.70

Values are mean ± SD of three determinations. N.D means not detected.

**Figure 1:**
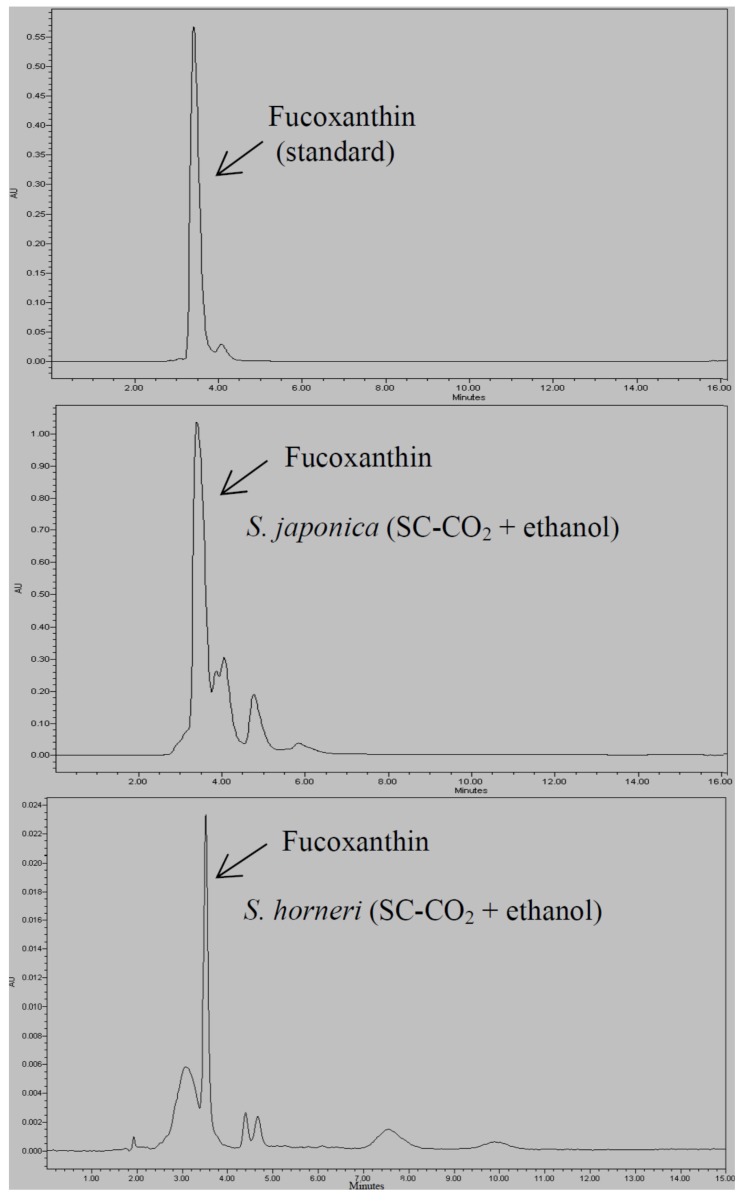
HPLC chromatogram of fucoxanthin content in *S. japonica* (SC-CO_2_ + ethanol) and *S. horneri* (SC-CO_2_ + ethanol) extracts.

The fucoxanthin contents in the brown seaweeds extracted by the various methods are shown in Table 1. The extracted oil from *S. horneri* by using SC-CO_2_ showed the highest content 0.77 ± 0.07 mg/g, followed by 0.71 ± 0.05 mg/g in the acetone–methanol extract. Moreover, the fucoxanthin in the *S. japonica* oil extracted by acetone–methanol, 0.48 ± 0.10 mg/g, was higher than that by SC-CO_2_ at 0.41 ± 0.05 mg/g. These results show that the SC-CO_2_ process can extract a similar content of fucoxanthin as when a solvent is used (Figure 1 and Supplementary Figure S1). A previous report on the fucoxanthin content in *S. horneri* noted 11.24 mg/g in methanol extract [21], and another reported 3.7 ± 1.6 mg/g DW in seaweed by using the same process [16]. In a third report, the fucoxanthin content in *S. japonica* by using ethanol was reported as 18.7 mg/100 g fresh weight [22]. Conde *et al.* [15] reported that 12 mg/100 g in *S. muticum* (SC-CO_2_ with 10% ethanol). The content of fucoxanthin was reported to vary significantly with season and life cycle of the algae, peaking between the winter and spring (mature phase of sporophyte) and lowest during summer (senescence phase) [16].

Carotenoids are widely present in marine biota, and their occurrence is species-dependent. Fucoxanthin is a dominant carotenoid characteristic of brown algae, particularly diatoms [22], whereas β-carotene, zeaxanthin and fucoxanthin are major pigments of red seaweed [16]. Fucoxanthin is the main carotenoid in various edible brown seaweed types such as *Undaria pinnatifida* and *Hijikia fusiforme* [23]. However, information on the content of fucoxanthin in the other brown seaweeds, edible or otherwise, is highly limited. The results of this study are important because several previous studies have established fucoxanthin as an anticancerous, antidiabetic, and antiobesity agent [16]. The results of our study indicate the potential of several brown types as sources of these multifunctional biomolecules.

### 2.4. Antioxidant Properties and Total Phenolic Content

#### 2.4.1. Hydrogen Peroxide Scavenging (H_2_O_2_) Assay

The yield of extracts from seaweed through SC-CO_2_ with ethanol was significantly higher than that when other extracts were used. The obtained results of all the extracts are shown in Table 3. The half maximal inhibitory concentration (IC_50_) value of SC-CO_2_ with ethanol extract from *S. horneri* was 686.31 ± 0.20 μg/mL, and that from *S. japonica* was 600.73 ± 0.15 μg/mL. The activities were found to be proportionately decreased when solvents such as hexane and ethanol were used. It is noted that acetone–methanol for both brown seaweeds was significantly effective in scavenging H_2_O_2_, with yields of 450.49 ± 1.42 μg/mL and 546.10 ± 1.05 μg/mL for *S. japonica* and *S. horneri*, respectively. The IC_50_ value of standard ascorbic acid was 448.19 ± 0.78 μg/mL.

Athiperumalsami *et al.* [24] reported that when using the H_2_O_2_ scavenging method, the methanoland water extracts of the seaweeds *Padina tetrastromatica*, *Ulva lactuca*, and *Acanthophora spicifera* showed the highest antioxidant activity, although the IC_50_ values were lower than that of standard tocopherol. The methanol extract of the seaweed *Gracilaria foliifera* had antioxidant activities higher than the standard tocopherol when tested by the H_2_O_2_ scavenging method. In the present study, similar results were obtained when ascorbic acid was used as a standard, and the SC-CO_2_ with ethanol extracts of seaweed showed higher values than the standard.

**Table 3:** Antioxidant activity, hydrogen peroxide scavenging assay, β-carotene linoleic acid method, total antioxidant activity and total phenolic content of brown seaweeds from various extracts.

	H_2_O_2_ IC_50_ (μg/mL)		β-carotene Bleaching Assay (%)		Total Antioxidant Capacity (mg/g α-tocopherol DW)		Total Phenolic Content (mg/g GAE DW)	
Solvent	*S. japonica*	*S. horneri*	*S. japonica*	*S. horneri*	*S. japonica*	*S. horneri*	*S. japonica*	*S. horneri*
SC-CO_2_ + ethanol	600.73 ± 0.15 ^a^	686.31 ± 0.20 ^a^	72.00 ± 0.74 ^a^	75.03 ± 1.56 ^a^	30.74 ± 0.13 ^a^	38.91 ± 3.79 ^a^	0.60 ± 0.05 ^a^	0.64 ± 0.01 ^a^
Acetone mix methanol	450.49 ± 1.42 ^b^	546.10 ± 1.05 ^b^	65.10 ± 3.03 ^b^	69.51 ± 0.35 ^b^	28.74 ± 1.38 ^b^	28.05 ± 0.72 ^b^	0.56 ± 0.04 ^b^	0.60 ± 0.01 ^b^
Hexane	100.15 ± 2.17 ^c^	125.18 ± 0.35 ^c^	31.81 ± 0.76 ^c^	36.58 ± 0.65 ^c^	16.67 ± 2.41 ^c^	19.77 ± 0.86 ^c^	0.28 ± 0.01 ^c^	0.42 ± 0.02 ^c^
Ethanol	200.02 ± 4.21 ^d^	220.62 ± 1.57 ^d^	42.66 ± 0.85 ^d^	46.71 ± 2.62 ^d^	22.5 ± 0.10 ^d^	25.63 ± 0.93 ^d^	0.34 ± 0.05 ^d^	0.43 ± 0.01 ^c^
Ascorbic acid	448.19 ± 0.78							
Trolox			84.56 ± 0.34					

Values are expressed as mean ± SD. Different letters indicate significant differences (*p* < 0.05) according to Tukey’s multiple range test.

**Figure 2:**
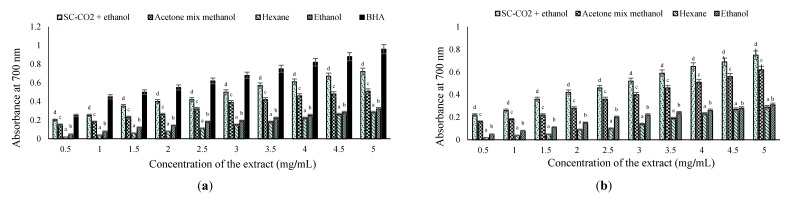
(**a**) Reducing power of *S. japonica* extracts with a standard antioxidant; (**b**) *S. horneri* extracts with a standard antioxidant. Error bars represent standard deviation with three replicates. Different letters indicate significant differences (*p* < 0.05) according to Tukey’s multiple range test.

#### 2.4.2. Reducing Power (RP) Method

The reducing powers of all of the extracts were increased at higher concentrations. In particular, the SC-CO_2_ with ethanol extract of *S. horneri* exhibited a reducing power effect as high as ascorbic acid at 5 mg/mL, transforming radical to non-radical by giving away hydrogen or an electron. The reducing powers of brown seaweed extracts were concentration-dependent (Figure 2a,b). As the concentration increased from 0.5 to 5.0 mg/mL, an increase in absorbance was noted for all of the solvents except hexane. However, the reducing powers of the samples were in the following order: butylated hydroxyanisole (BHA) (0.25–0.96) > SC-CO_2_ with ethanol extract of *S. horneri* (0.22–0.74) > SC-CO_2_ with ethanol extract of *S. japonica* (0.20–0.70) > acetone–methanol extract of *S. horneri* (0.16–0.62) > acetone–methanol extract of *S. japonica* (0.15–0.53) > ethanol extract of *S. horneri* (0.05–0.31) > ethanol extract of *S. japonica* (0.05–0.30) > hexane extract of *S. horneri* (0.02–0.29) > hexane extract of *S. japonica* (0.02–0.28). The reducing power increased with an increase in concentration in all of the samples. The same trend was also reported by Kumaran *et al.* [25] in the methanolic extracts of higher plants. All concentrations exhibited an optical density (OD) value of <1.0. This property is associated with the presence of reductones that are reported to be terminators of the free radical chain reaction [26].

#### 2.4.3. β-Carotene Linoleic Acid Method

The antioxidant activities of the seaweed extracts through β-carotene bleaching assay are shown in Table 3. Among all tested extracts, only SC-CO_2_ with ethanol showed good activity against this system, with *S. japonica* at 72.00% ± 0.74% and *S. horneri* at 75.03% ± 1.56%. The standard (Trolox) showed 84.56% ± 0.34%. Many tests have been developed to evaluate the antioxidant activity of various samples to account for their chemical diversity. Some determine the ability of antioxidants in scavenging free radicals generated in the reaction medium, such as DPPH (2,2-diphenyl-1-picrylhydrazyl); others measure the inhibition of lipid oxidation through the antioxidant system to be tested, such as β-carotene. Therefore, it is necessary to evaluate the effect of the methodology used.

The mechanism of bleaching of β-carotene is a free radical-mediated phenomenon resulting from the H_2_O_2_ formed from linoleic acid. The β-carotene in this model system undergoes rapid discoloration in the absence of an antioxidant. The linoleic acid free radical attacks the highly unsaturated β-carotene molecules. As β-carotene molecules lose their double bonds by oxidation, the compound loses its chromophore and characteristic orange color, which can be monitored spectrophotometrically; that is, the absorbance values decrease with time as indicated by the rate of peroxidation [27]. The presence of an antioxidant can hinder the extent of β-carotene bleaching by neutralizing the linoleate free radical and other free radicals formed in this system; consequently, the absorbance values show different decreasing patterns. Generally, stronger antioxidant activity relate to slower decreases in absorbance values with time [28].

In the β-carotene assay, the oxidation of linoleic acid generates peroxyl free radicals due to the abstraction of hydrogen atoms from diallylic methylene groups of linoleic acid [29]. The free radical then will oxidize the highly unsaturated β-carotene. Consequently, the orange-colored chromophore of β-carotene would be degraded, and the result could be monitored spectrophotometrically. However, the presence of antioxidant constituents could neutralize the linoleate free radical and hence prevent the bleaching of β-carotene [28].

#### 2.4.4. Total Antioxidant Activity

The global antioxidant activities of extracts were expressed as the number of α-tocopherol equivalents. The phosphomolybdenum method was based on the reduction of Mo (VI) to Mo (V) by the antioxidant compound and the formation of a green phosphate/Mo (V) complex. The antioxidant activity of *S. horneri* was higher than that of *S. japonica* extracts (Table 3). Higher activity of 38.91 ± 3.79 and 30.74 ± 0.13 mg/g α-tocopherol DW was observed in SC-CO_2_ with ethanol extract of *S. horneri* and *S. japonica*, respectively. The acetone–methanol extract also showed 28.05 ± 0.72 and 28.74 ± 1.38 mg/g α-tocopherol DW of *S. horneri* and *S. japonica*, respectively.

Chandini *et al.* [25] have reported total antioxidant activity in the range of 245 to 376 mg ascorbic acid equivalents/g in the case of brown seaweed extracts. Higher activity was observed in fractions as compared to total methanolic extract. Higher activity in fractions may be due to the interferences of other compounds present in crude (methanolic) extract; and, it has also been reported that solvents used for extraction have dramatic effect on the chemical species [30]. It has been earlier reported that some major active compounds from brown seaweed that have antioxidative properties are phlorotannins and fucoxanthin. Also, antioxidative activities of brown seaweeds cannot be attributed to their characteristic pigment (fucoxanthin) or any other carotenoids alone [25].

#### 2.4.5. Total Phenolic Content

Phenolic compounds are widely distributed in the plant kingdom and have been reported to have several biological activities including antioxidant properties. Earlier reports revealed that marine seaweed extracts, particular their polyphenols, have antioxidant activity. Phlorotannins and fucoxanthin have been reported as major active compounds in various seaweed extracts [31]. Previous studies have shown that phenolic compounds are the main contributors to the antioxidant activity of various seaweeds [32]. The total phenolic content (TPC) of the seaweed extracts was calculated by using a modified Folin-Ciocalteu method (Table 3). The oil of *S. japonica* extracted with SC-CO_2_ and ethanol and that of *S. horneri* , at 0.60 ± 0.05 mg/g and 0.64 ± 0.01 mg/g GAE DW, respectively, demonstrated higher TPC than that in all other seaweed-extracted oils. The acetone–methanol-extracted oil demonstrated high levels of phenolic in the lipid extraction from *S. japonica* and *S. horneri* at 0.56 ± 0.02 and 0.60 ± 0.01 mg/g GAE DW, respectively.

The concentration of polyphenols in seaweed depends on many variables such as habitat, season of harvesting and environmental conditions including light, temperature, and salinity. In addition, the distribution of phenols varies among species [33]. Brown seaweeds showed higher total polyphenol contents than those in red seaweeds. Among the species studied, two *Fucales*, *Bifurcaria bifurcata* and *Himantalia elongata*, displayed the highest polyphenol contents. This feature is in agreement with previous studies of some Phaeophyceae from Brittany coasts [34] that report Fucales as those among brown seaweeds with the highest polyphenol content. In addition, higher polyphenol contents have been reported in brown seaweeds than those in red [35]. The organic solvent was more efficient than aqueous extraction for polyphenolic compounds in all species tested. This result is in agreement with those in previous studies reporting the aqueous mixtures of methanol, ethanol, or acetone as more effective extractants of polyphenol compounds [36,37,38].

### 2.5. Antimicrobial Studies

To examine the antimicrobial activity of seaweed extracts obtained from various solvent extracts, we tested three gram-positive bacteria, *Listeria monocytogenes*, *Bacillus cereus*, and *Staphylococcus aureus*; three gram-negative bacteria, *Escherichia coli*, Pseudomonas *aeruginosa*, and Salmonella *typhimurium*; and two fungi, *Candida albicans* and *Aspergillus* *brasiliensis* (Table 4). Among the different seaweeds extracts, the exhibited antimicrobial activity against *E. coli* and *S. aureus*, at 28 ± 1.50 and 24 ± 0.10 mm, respectively, was the highest in the extract of acetone–methanol of *S. horneri*, followed by the acetone–methanol extract of *S. japonica* against *E. coli* and *S. aureus*, at 21 ± 0.50 and 20 ± 0.50 mm, respectively. No activity was found against *P. aeruginosa* and *S. typhimurium*. The SC-CO_2_ with ethanol extract also showed good activity similar to the solvent extractions. The SC-CO_2_ with ethanol extract of *S. japonica* showed good antimicrobial activity against *E. coli* at 18 ± 0.10 mm, *L. monocytogenes* at 12 ± 0.05 mm, *B. cereus* at 10 ± 0.01 mm, *S. aureus* at 18 ± 0.50 mm, *C. albicans* at 12 ± 0.20 mm, and *A. brasiliensis* at 16 ± 0.00 mm. The SC-CO_2_ with ethanol extract of *S. horneri* showed good antimicrobial activity against *E. coli* at 21 ± 1.10 mm, *L. monocytogenes* at 14 ± 0.20 mm, *B. cereus* at 12 ± 0.60 mm, *S. aureus* at 20 ± 0.45 mm, *C. albicans* at 16 ± 0.80 mm, and *A. brasiliensis* at 18 ± 0.50 mm). These results clearly show that the *S. horneri* extract had a higher amount of activity than that of *S. japonica*. The acetone–methanol showed more activity than the SC-CO_2_–ethanol extracts.

**Table 4:** Antimicrobial activity of *S. japonica* and *S. horneri* from various extracts by the diameters of the inhibition zones (mm) using the disc diffusion method.

	Inhibition Zone (mm)							
Microorganisms	*S. japonica* extracts				*S. horneri* extracts			
	SC-CO_2_ + Ethanol	Acetone mix Methanol	Hexane	Ethanol	SC-CO_2_ + Ethanol	Acetone Mix Methanol	Hexane	Ethanol
*E. coli*	18 ± 0.10 ^d^	21 ± 0.50 ^e^	15 ± 0.10 ^b^	16 ± 0.70 ^c^	21 ± 1.10 ^f^	28 ± 1.50 ^c^	18 ± 0.90 ^c^	19 ± 0.90 ^c^
*P. aeruginosa*	N.D	N.D	N.D	N.D	N.D	N.D	N.D	N.D
*S. typhimurium*	N.D	N.D	N.D	N.D	N.D	N.D	N.D	N.D
*L. monocytogenes*	12 ± 0.05 ^b^	15 ± 0.20 ^a^	8 ± 0.25 ^a^	9 ± 0.08 ^b^	14 ± 0.20 ^b^	24 ± 0.10 ^b^	8 ± 0.02 ^a^	10 ± 0.02 ^b^
*B. cereus*	10 ± 0.01 ^a^	16 ± 0.60 ^b^	N.D	N.D	12 ± 0.60 ^a^	17 ± 0.30 ^a^	N.D	N.D
*S. aureus*	18 ± 0.50 ^d^	20 ± 0.50 ^d^	8 ± 0.40 ^a^	6 ± 0.05 ^a^	20 ± 0.45 ^e^	24 ± 0.10 ^b^	10 ± 0.15 ^b^	7 ± 0.04 ^a^
*C. albicans*	12 ± 0.20 ^b^	18 ± 0.10 ^c^	N.D	N.D	16 ± 0.80 ^c^	24 ± 0.55 ^b^	N.D	N.D
*A. brasiliensis*	16 ± 0.00 ^c^	20 ± 0.10 ^d^	N.D	N.D	18 ± 0.50 ^d^	28 ± 0.85 ^c^	N.D	N.D

Values are expressed as mean ± SD. N.D means not detected. Different letters indicate significant differences (*p* < 0.05) according to Tukey’s multiple range test.

Gaurav *et al.* [39] report the antimicrobial activity of various methanolic extracts of *H. elongate* by using the disc diffusion method. Various food spoilage bacteria such as *E. faecalis* and *P. aeruginosa* and pathogenic bacteria such as *L. monocytogenes* and *S. abony* were used to determine the antibacterial activities of brown seaweed aqueous methanolic extracts and synthetic compounds by disc diffusion assay. The zone of inhibition of seaweed extracts was measured by using the reference of the inhibition exhibited by synthetic food antimicrobials. The antimicrobial activity in our studied seaweed was significantly higher than that reported in the previous study [39].

### 2.6. Antihypertensive Activity

In this study, the extracts of *S. japonica* and *S. horneri* were screened for their potential ACE-I activities, as shown in Table 5. The acetone mix methanol extract of *S. horneri* had the maximum IC_50_ value at 1.28 ± 0.50 μg/mL, whereas that of the SC-CO_2_–ethanol extract was 0.97 ± 0.11 μg/mL. *S. japonica* also exhibited significant activity in acetone–methanol extract, with an IC_50_ value of 1.05 ± 0.14 μg/mL. That in the SC-CO_2_–ethanol extract was 0.89 ± 0.07 μg/mL, respectively, whereas hexane and ethanol exhibited weak activities. *S. horneri* extracts showed good ACE-I activity compared with those of *S. japonica* extracts. Therefore, our two brown seaweeds extracts exhibited moderate ACE-I activity. Katsumi *et al.* [40] reported that fucoxanthin isolated from *Undaria pinnatifida* has been shown to lessen the development of hypertension and its related diseases in stroke-prone spontaneously hypertensive rats. So, the above ACE-I activity could be attributed to the fucoxanthin content in the crude extracts of both brown seaweed.

Jung *et al.* [41] evaluated the ACE-I activity of some brown seaweeds using ethanol extracts. In his report, except *Hizikia fusiforme*, all of the brown algal crude extracts showed favorable ACE-I activity, particularly *Ecklonia stolonifera Okamura* and *Ecklonia cava* *Kjellman* at 64.86% and 166.67%, respectively, at a concentration of 163.93 mg/mL. Cha *et al.* [42] performed a study to screen *in vitro* ACE-I activities of separate methanol and aqueous prepared from 26 macro algae obtained from the Coast of Jeju Island in Korea. The macroalgal extracts with the most impressive ACE-I activities were the aqueous extracts of *Lithophyllum okamurae* Foslie and *Lomentaria catenata* with IC_50_ values of 13.78 and 12.21 mg/mL, respectively, and the methanol extract from *Ahnfeltiopsis flabelliformis* with an IC_50_ value of 13.84 mg/mL [42].

**Table 5:** Antihypertensive activity of *S. japonica* and *S. horneri* from various extracts.

	IC_50_ (μg/mL)	
Solvent	*S. japonica* Extracts	*S. horneri* Extracts
SC-CO_2_ + ethanol	0.89 ± 0.07 ^b^	0.97 ± 0.11 ^b^
Acetone mix methanol	1.05 ± 0.14 ^a^	1.28 ± 0.50 ^a^
Hexane	0.03 ± 0.01 ^c^	0.01 ± 0.00 ^c^
Ethanol	0.05 ± 0.01 ^c^	0.07 ± 0.01 ^c^
Captopril	0.08 ± 0.02 μg/mL	

Values are expressed as mean ± SD. Different letters indicate significant differences (*p* < 0.05) according to Tukey’s multiple range test.

## 3. Experimental Section

### 3.1. Materials

During June 2013, *S. horneri* (Turner), C.Agardh, 1820, was collected from the seacoast in the southern part of Korea, and *S. japonica*, J.E. Areschoug, 1851, Lane, Mayes, Druehl, and Saunders was collected from Guemil-eup, Wando-gun, Jeollanam-do, Korea. High-purity CO_2_ gas (99%) was supplied by KOSEM (Yangsan, Korea). Fucoxanthin, Captopril, *O*-phthalaldehyde (OPA), hippuryl histidyl leucine (HHL), BHA, gallic acid, Trolox, H_2_O_2_, α-tocopherol, ascorbic acid, β-carotene, Folin-Ciocalteu reagent, Mueller-Hinton agar, and dimethyl sulphoxide (DMSO) were purchased from Sigma-Aldrich Chemical Co. (St. Louis, MI, USA). All of the reagents used in this study are analytical or high-performance liquid chromatography (HPLC) grade.

### 3.2. Sample Preparation

The fresh *S. horneri* and *S. japonica* samples were washed by using distilled water, and the samples were then chopped into small pieces. They were next dried by using a freeze drier (Eyela FDU-2100, Tokyo Rikakikai Co., Ltd., Tokyo, Japan) equipped with a square-type drying chamber (Eyela DRC-1000, Tokyo Rikakikai Co., Ltd., Tokyo, Japan) at a temperature of −80 °C for 72 h. The freeze-dried seaweed samples were then collected, finely ground by using a mechanical blender (PN SMKA-4000 mixer, PN Co., Ltd., Ansan-si, Korea), and sieved by using a 710 μm stainless steel sieving mesh. Samples that passed though the sieving mesh were stored at −20 °C for one day prior to use.

### 3.3. Conventional Solvent Extraction

Seaweed powder were contacted with hexane, ethanol, acetone mixed methanol (1:1, v/v) at a liquid of solid ratio of 20:1 (v/w) at 25 °C for 300 rpm using magnetic stirrer for 20 h protected from light to limit the possibility of oxidation. Solids were separated by filtration through Whatman No.1 filter paper and liquid solutions were evaporated in a rotary vacuum evaporator (EYELA N-1100, Tokyo, Japan) at 40 °C. The oils were stored at 4 °C until further use and analysis.

### 3.4. Supercritical Fluid Extraction with Carbon Dioxide

**Figure 3:**
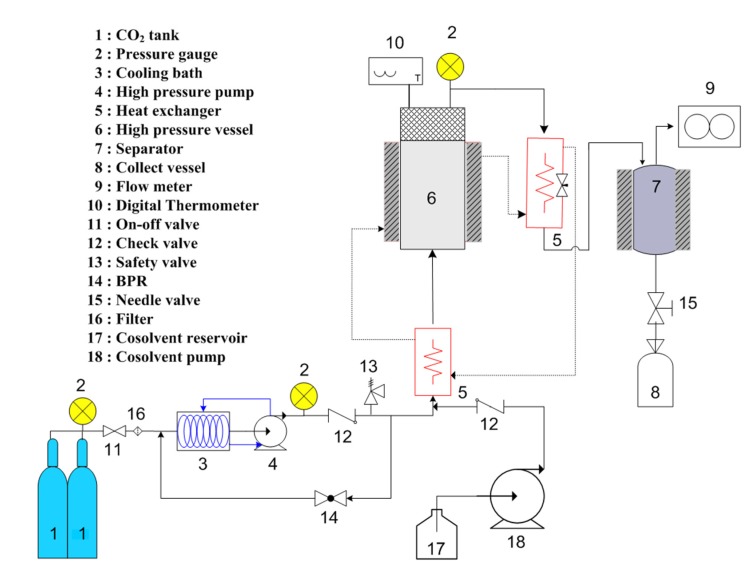
Schematic diagram of supercritical fluid extraction with ethanol as co-solvent.

A laboratory scale of the SC-CO_2_ process is shown in Figure 3. Exactly 100 g of freeze dried seaweed powder was filled into a 200 mL stainless steel extraction vessel. A thin layer of cotton was placed at the bottom of the extraction vessel and on the top of the sample, and the extraction vessel was then plugged with a cap. A high-pressure pump (Milroyal; Milton Roy, PA, USA) was applied to pump liquid CO_2_ into the extraction vessel to reach the desired pressure. The CO_2_ pressure was controlled by a back pressure regulator. A water bath connected to the extraction vessel was used to maintain the temperature. A gas flow meter (Shinagawa, DC-1, Tokyo, Japan) was used to measure the CO_2_ consumed during the extraction period. The seaweeds were studied at a temperature of 45 °C and pressure of 250 bar for oil extraction. The CO_2_ flow rate was constant at 27 g/min during the entire extraction period of 2 h. Ethanol (96%) was used as a co-solvent with a flow rate of 1 mL/min. Finally, the extracted oil was collected from the separating vessel, and the ethanol was removed in a rotary vacuum evaporator (EYELA N-1100, Tokyo, Japan) at 40 °C before being stored at 4 °C until further use and analysis.

### 3.5. Fucoxanthin Analysis by HPLC

The HPLC analysis was conducted by using a Waters 600 E HPLC system (Waters, Milford, MA, USA) equipped with a tunable absorbance detector (Waters, Milford, MA, USA). The fucoxanthin was analyzed following the method of Roh *et al.* [43] with minor modification. The fucoxanthin content in the seaweed extracts was determined by reversed-phase HPLC (RP-HPLC) with methanol–acetonitrile (7:3, v/v) as the mobile phase at a flow rate of 1.0 mL/min. All RP-HPLC analysis was conducted at ambient temperature by using a RP column (XTerra^®^ MS C_18_) with a 5.0 μm particle size and 250 mm × 4.6 mm inner diameter (id; Waters, Milford, MA, USA) protected by a guard column of 10 mm × 4.6 mm id with the same stationary phase. Briefly, an aliquot of seaweed extract was dissolved in a mobile phase and was then filtered with a 0.22 μm membrane filter. The fucoxanthin detection wavelength was set at 450 nm. A standard curve prepared by using the authentic standard was used for quantification of the fucoxanthin content in the seaweed samples. The fucoxanthin content in the seaweed sample was expressed as milligrams per gram. The amount of fucoxanthin was quantified from the peak area by using a standard curve with commercial fucoxanthin (Sigma-Aldrich, St. Louis, MI, USA).

### 3.6. FA Composition Analysis

FA compositions of seaweed obtained after SC-CO2 and organic solvent extraction were analyzed by GC following the method of Lee *et al.* [44]. A 6890 Agilent Technologies (Wilmington, DE, USA) gas chromatograph was used with a fused silica capillary column of 100 m in length, 0.25 mm in i.d., and film length of 0.2 μm (Supelco, Bellefonte, PA, USA). Before running the sample in the GC, the sample was prepared by using methyl ester according to the official method and recommended practices of the American Oil Chemists’ Society (AOCS) [45]. The flow rate of Helium as a carrier gas was 1.0 mL/min. The oven temperature was programmed to begin at 130 °C constant temperature for 3 min, increase to 240 °C at a rate of 4 °C min^−1^, and hold at 240 °C for 10 min. The injector and detector temperatures were both 250 °C. FA methyl esters were identified by comparison of retention time with standard FA methyl esters mixture (Supelco, Bellefonte, PA, USA).

### 3.7. Antioxidant Activity Assessment

#### 3.7.1. H_2_O_2_ Scavenging Assay

Hydroxyl radical scavenging activity was analyzed according to Xia *et al.* [46]. A 40 mM solution of H_2_O_2_ was prepared in 50 mM of a phosphate buffer at pH 7.4. The concentration of H_2_O_2_ was determined by absorption at 230 nm using a spectrophotometer (UVmini-1240, Shimadzu Co., Kyoto, Japan). 20–60 μg/mL of seaweed extract was added to H_2_O_2_, and absorbance at 230 nm was determined after 10 min against a blank solution of methanol containing a phosphate buffer without H_2_O_2_. 0.1 M of ascorbic acid was used as standard. The percentage of H_2_O_2_ scavenging was calculated as

% Scavenged (H_2_O_2_) = [(*A*_i_ − *A*_t_)/*A*_i_] × 100 (1)

where *A*_i_ is the absorbance of the control, and *A*_t_ is the absorbance of the test or standard. The IC_50_ value (mg/mL) is the concentration of the sample or standard required to scavenge 50% of H_2_O_2_ free radicals.

#### 3.7.2. RP Method

The reducing power of the seaweed extracts was determined following Deng *et al.* [47] with minor modifications. Briefly, 1 mL of seaweed extract solution with various concentrations of 0.5–5 mg/mL was mixed with 0.2 mL of 2 M sodium phosphate buffer (pH 6.6) and 0.5 mL 1% (w/v) aqueous potassium ferricyanide. The mixture was incubated at 50 °C for 20 min in a water bath. Then, 2.5 mL of 10% (w/v) of trichloroacetic acid was added to the mixture. The resultant mixture was centrifuged at 3500 rpm for 10 min. Two milliliters of the supernatant was diluted with 3 mL distilled water and was then mixed with 0.5 mL of 0.3% (w/v) ferric chloride. The absorbance was measured at 700 nm against distilled water. The increase in absorbance indicated an increase in reducing power. The sample blank (methanol) and control (0.5 mg/mL of standard BHA) sample analysis was also performed according to the method of Deng *et al.* [47].

#### 3.7.3. β-Carotene Linoleic Acid Method/Conjugated Diene Assay

The β-carotene linoleic acid method was performed following the method of Wang *et al.* [48] with some modifications. Briefly, oxygenated water was mixed with β-carotene, Tween™ 40, and linoleic acid. Two hundred microliters of the mixture was incubated with 10 mg/mL of seaweed extracts, and 50 μL was incubated without seaweed extracts for 120 min at 50 °C. The absorbance was read at 450 nm (UVmini 1240, Shimadzu Co., Kyoto, Japan) at time 0 and every 30 min thereafter. The results are expressed as percentage of β-carotene bleaching inhibition. The sample blank (methanol) and Trolox (0.5 mg/mL) were used as standards, and antioxidant activity was calculated as a percentage of inhibition (*I*%) relative to the control by using the following equation:

*I*% = [1 − (*A*_s_ − *A*_s120_)/*A*_c_ − *A*_c120_)] (2)

where *A*_s_ is initial absorbance, *A*_s120_ is the absorbance of the sample at 120 min, *A*_c_ is the initial absorbance of the control, and *A*_c120_ is the absorbance of the control at 120 min.

#### 3.7.4. Total Antioxidant Activity

The total antioxidant activity assay was based on the reduction of Mo (VI) to Mo (V) by the sample analyses and subsequent formation of a green phosphate Mo (V) complex at acidic pH. The total antioxidant capacity can be calculated by the method described by Pan *et al.* [49]. Then, 0.1 mL of 100 μg/mL seaweed extract solution was combined with 1 mL of reagent including 0.6 M sulfuric acid, 28 mM sodium phosphate, and 4 mM ammonium molybdate. The tube was capped and incubated in a boiling water bath at 95 °C for 90 min. After cooling the sample to room temperature, the absorbance of the aqueous solution was measured at 695 nm against the blank by using the UV spectrophotometer. A typical blank solution contained 1 mL of the reagent solution and the appropriate volume of the same solvent used for the sample. The solution was incubated under the same conditions as those for rest of the sample. For samples of unknown composition, the antioxidant capacity was expressed as equivalents of α-tocopherol.

### 3.8. TPC Assay

The TPC of the seaweed extracts was determined by using the Folin-Ciocalteu colorimetric method as described by Li *et al.* [50] with minor modification. 1 mL of 1 g/10 mL seaweed extract was mixed 1 mL of 1:10 (v/v, in deionized water) diluted Folin–Ciocalteu reagent (FCR). After 4 min, 800 μL of 7.5% sodium carbonate solution (w/v) was added into the mixture. Then, the mixture was vortexed for 5 s and stored at room temperature in a dark environment for 2 h. The blank was also prepared by replacing 1 mL of the deionized water. The absorbance of the mixture was measured at 765 nm against the blank by using the UV-spectrophotometer. The measurements were performed in triplicate. Gallic acid was used for calibration of the standard curve (*y* = 0.000653*x* + 0.072). The results are expressed in Gallic acid equivalents (GAE).

### 3.9. Antimicrobial Activity

Six bacterial strains of food-borne pathogens were used in this study including three gram-negative bacteria, *E. coli* ATCC 25922, *P. aeruginosa* ATCC 9027, and *S. typhimurium* KCCM 11862; three gram-positive bacteria, *S. aureus* ATCC 6538p, *L. monocytogenes* ATCC 7644, and *B. cereus* ATCC 13061 in addition two fungal strains, *C. albicans* ATCC 10231 and *A. brasiliensis* ATCC 16404. The microbial strains were obtained from the Korean Culture Center of Microorganisms, Republic of Korea. The antimicrobial activities were determined by using the agar diffusion method following Meillisa *et al.* [51] with modifications. McFarland standard No. 0.5 was used in the preparation of microorganism suspension. The turbidity of the bacterial suspension was adjusted according to the McFarland standard. Accurate turbidity of the bacterial suspension was confirmed by spectrophotometry at 625 nm, with the approximate cell density of each bacterial strain at 10^7^ CFU/mL. Mueller-Hinton agar (Sigma-Aldrich, Bengaluru, India), was used as a growth medium and was sterilized at 121 °C for 15 min. The agar was poured into sterile glass Petri dishes and was allowed to set, and the bacterial suspension was spread on the agar surface with sterile cotton. An Advantec paper disk containing 10 µL (100 μg /mL) seaweed extract was diluted by using DMSO and 10 μL of DMSO to serve as a negative control, which was placed on the surface of the agar. The plates were incubated at 37 °C for 24 to 48 h, and the antimicrobial activity was identified by measuring the diameter of the clear zone.

### 3.10. Determination of Antihypertensive Activity

Antihypertensive activity was detected by using Angiotensin I-Converting Enzyme (ACE) inhibitory assay following Ko *et al.* with slight modifications [52]. Buffer A, pH 8.3 with HCl, contained 20 mM sodium borate and 0.3 M NaCl. Buffer B, pH 12.0 with NaOH, was composed of 0.1 M sodium borate and 0.2 M NaOH. The OPA reagent, composed of Fluoraldehyde Reagent Solution, was prepared at least 1 h prior to performing the experiment by mixing 1.5 mL of 10 mg/mL OPA solution in ethanol and 1.5 mL of 5 µL/mL of 2-mercaptoethanol solution in ethanol in 100 mL of Buffer B. The final concentrations of OPA and 2-mercaptoethanol in the OPA reagent were both approximately 1 mM. The ACE and HHL solutions were freshly prepared by dissolving 40 mU/mL ACE and 15 mM HHL with Buffer A, respectively.

Seaweed extracts were diluted to various extents of 0.1–5 mg/mL with 0.1% DMSO. The ACE catalyzed reactions (37 °C × 2 h) were performed in test tubes containing 100 µL of seaweed extract, 100 µL of ACE solution, and 100 µL of HHL solution (Mixture 1; M1). A second mixture containing 100 µL of sample solution and 200 µL of Buffer A (Mixture 2; M2) was used to obtain the background absorbance of the sample solutions for the colorimetric method. A third mixture containing 100 µL of Buffer A, 100 µL of ACE solution, and 100 µL of HHL solution (Mixture 3; M3) was used to obtain the data for 100% reaction. A fourth mixture containing 300 µL of Buffer A (Mixture 4; M4) was used to obtain the background absorbance of the OPA reagent. The enzymatic reactions at working pH levels of 5–10 were terminated by adding 3 mL of the alkaline (pH 12.0). All the mixtures were measured at 390 nm after 20 min of 25 °C incubation. The inhibitory ratios were calculated by the following equation:

*I* (%) = [1 − (M1 − M2)/(M3 − M4)] × 100 (3)

The IC_50_ value is defined as the concentration of inhibitor required for 50% of ACE-I activity. Captopril was used as the standard.

### 3.11. Statistical Analysis

All mean values were analyzed by one-way analysis of variance (ANOVA). Values are expressed as mean ± standard deviation. (SD) with three replicates (SPSS software, Version 20 for Windows, IBM, Chicago, IL, USA). Statistical analysis was performed by using the Tukey method; *p* < 0.05 was considered to be significant. The IC_50_ concentration was calculated by using BioToolKit 320 trial version.

## 4. Conclusions

In this study, SC-CO_2_ extraction proved successful for extracting high yields of oil, FAs, phenolic compounds, and fucoxanthin content from brown seaweeds *S. horneri* and *S. japonica* when compared with other solvent extraction processes. The extracted oil from SC-CO_2_ also showed strong antioxidants, phenolics, antimicrobial and antihypertensive activities. The extracted oil from *S. horneri* by SC-CO_2_ showed high contents of fucoxanthin and also had better biological activities than that of *S. japonica*. Therefore, the obtained oil, with a rich content of bioactive materials, can be used in foods, pharmaceuticals, and cosmetics. Further research is underway for the purification of fucoxanthin from the brown seaweeds by using environmentally friendly SC-CO_2_ extraction.

## Supplementary Files

Supplementary File 1
