# Supplementary material for: Biological Properties of Fucoxanthin in Oil Recovered from Two Brown Seaweeds Using Supercritical CO2 Extraction
Source: Mar Drugs. 2015 May 29;13(6):3422–42. doi: 10.3390/md13063422 (PMC4483637; doi:10.3390/md13063422)
Supplement: Supplementary File 1 [file marinedrugs-13-03422-s001.pdf]

Supplementary Information

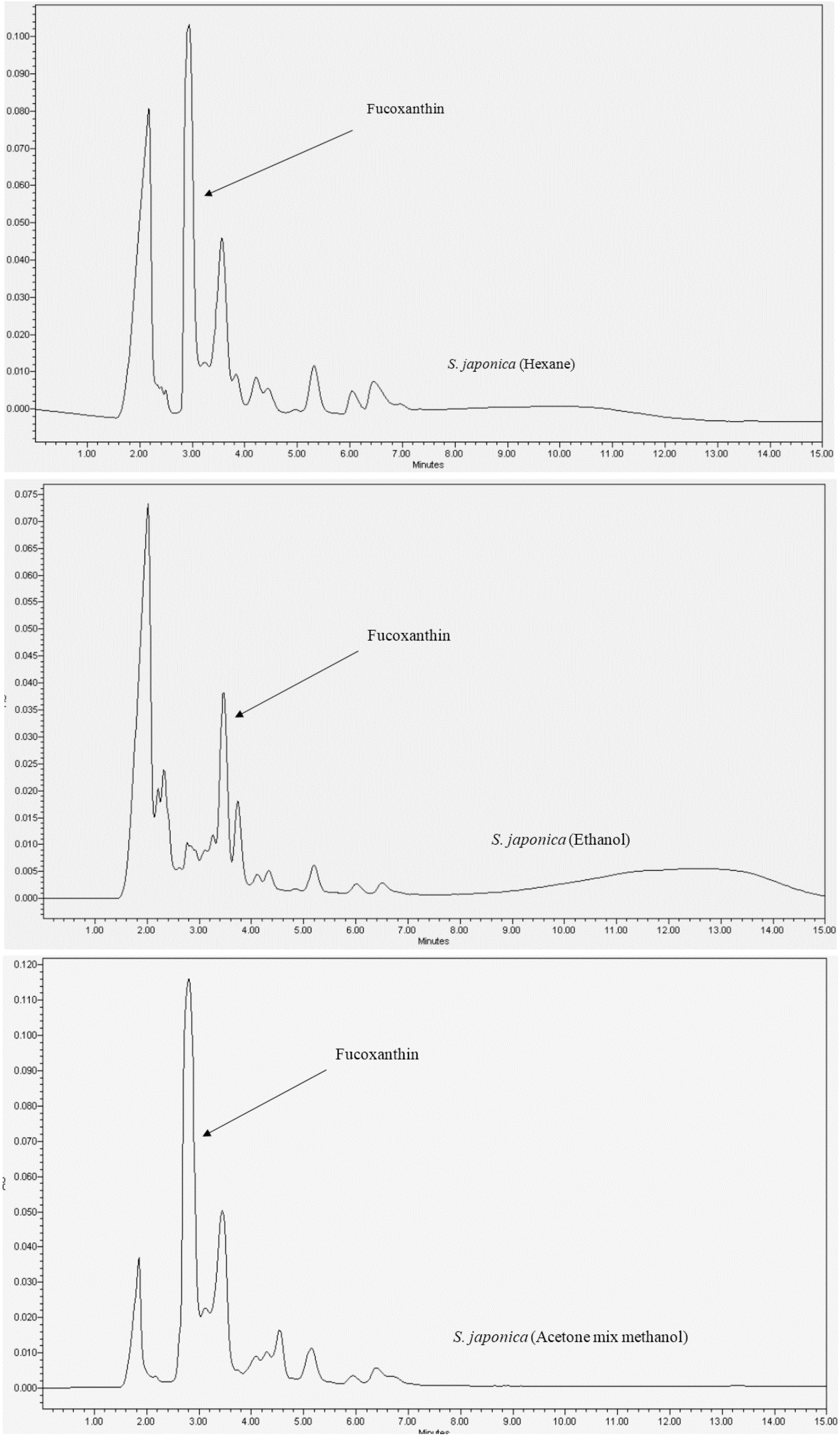

(a)

Figure S1. *Cont.*

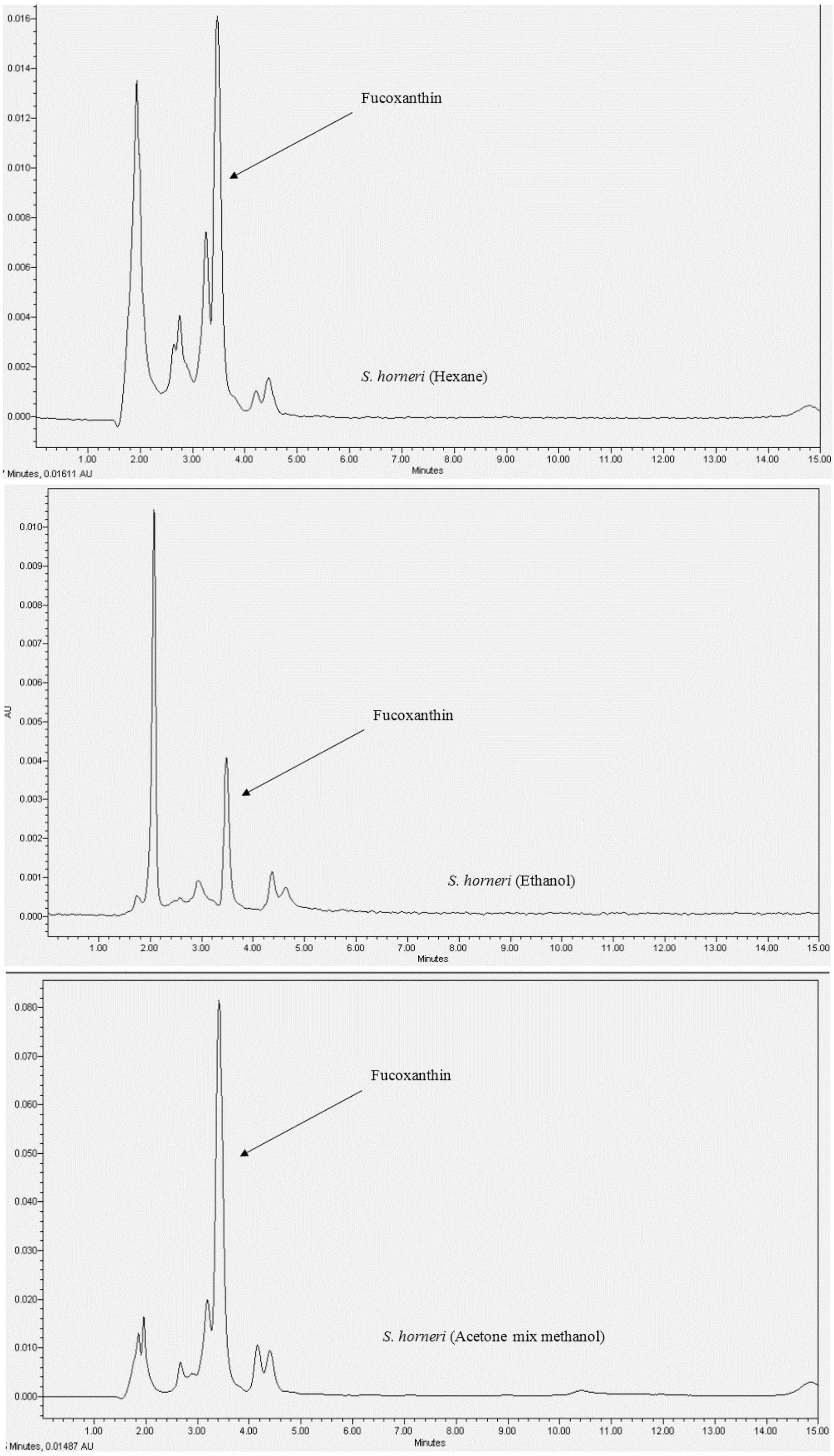

(**b**)

Figure S1. HPLC chromatogram of fucoxanthin content in (**a**) *S. japonica* using various solvent extraction and (**b**) *S. horneri* using various solvent extraction.

© 2015 by the authors; licensee MDPI, Basel, Switzerland. This article is an open access article distributed under the terms and conditions of the Creative Commons Attribution license (http://creativecommons.org/licenses/by/4.0/).
